# Optogenetics: A Novel Therapeutic Avenue for Age-Related Macular Degeneration

**DOI:** 10.3390/biom15091286

**Published:** 2025-09-05

**Authors:** Pier Luigi Grenga, Chiara Ciancimino, Alessandro Meduri, Serena Fragiotta

**Affiliations:** 1 UOC Ophthalmology, G.B. Grassi Hospital, Via Gian Carlo Passeroni, 28, Ostia, 00122 Rome, Italy; pierluigi.grenga@aslroma3.it; 2 Ophthalmology Unit, NESMOS Department, St. Andrea Hospital, “Sapienza” University of Rome, Via di Grottarossa 1035/1039, 00189 Rome, Italy; chiara.ciancimino@uniroma1.it; 3 Ophthalmology Clinic, Department of Biomedical Sciences, University of Messina, 98122 Messina, Italy; alessandro.meduri@unime.it

**Keywords:** age-related macular degeneration, optogenetics, treatment, adeno-associated viruses (AAVs)

## Abstract

Age-related macular degeneration (AMD) is a leading cause of irreversible vision loss in the elderly, characterized by progressive degeneration of the retinal pigment epithelium (RPE) and photoreceptors in the macula. Current treatment options primarily focus on slowing disease progression in neovascular AMD, while effective therapies for dry AMD remain limited. Optogenetics, a revolutionary technique utilizing light-sensitive proteins (opsins) to control the activity of genetically targeted cells, has emerged as a promising therapeutic strategy for restoring vision in retinal degenerative diseases. In retinal disease models, adeno-associated viruses (AAVs) serve as delivery vectors via intravitreal or subretinal injections. This review explores the principles of optogenetics, its application in preclinical AMD models, and the potential for clinical translation of this approach. We discuss the various optogenetic tools, delivery methods, and the challenges and future directions in harnessing this technology to combat AMD-related vision loss.

## 1. Introduction

Age-related macular degeneration (AMD) is a significant global health burden, contributing to blindness in approximately 5.6% of the global population. The prevalence of AMD affected 200 million people in 2020 and is projected to rise to nearly 300 million by 2040, posing substantial social and economic challenges [1,2]. AMD primarily affects individuals over the age of 55, threatening high-acuity central vision necessary for essential activities such as reading, driving, and facial recognition [3,4].

The disease initially manifests with extracellular deposits, seen ophthalmoscopically as drusen. Late-stage complications can occur in two primary forms: dry AMD or geographic atrophy (GA), characterized by atrophy of the retinal pigment epithelium (RPE) and photoreceptors, and wet or exudative AMD, marked by choroidal neovascularization, leading to fluid leakage and retinal damage due to fibrosis and photoreceptor loss [5].

AMD progresses through four distinct pathological stages: Stage 1 (Normal aging)—Minimal drusen deposits (<63 μm) without detectable RPE cell anomalies; stage 2 (Early AMD)—Intermediate-sized drusen (63–124 μm) without significant RPE irregularities; stage 3 (Intermediate AMD)—Large drusen (>125 μm), often associated with pigmentary abnormalities; and stage 4 (Advanced AMD)—Geographic atrophy (GA) affecting the fovea and neovascular maculopathy, both contributing to significant vision loss [6,7,8].

GA is the advanced form of dry AMD, characterized by progressive and irreversible vision loss resulting from outer retina and RPE atrophy. While early AMD is typically asymptomatic or associated with only minor visual disturbances, intermediate AMD may present clinically with difficulties in reading or the development of a central scotoma. Advanced AMD leads to profound and irreversible central vision impairment, severely impacting daily life [9]. This has driven the development of therapeutic strategies aimed at controlling late-stage complications, with the ultimate goal of preventing their onset.

Anti-angiogenic therapies have transformed the treatment of neovascular AMD, providing substantial improvements in visual outcomes. By targeting vascular endothelial growth factor (VEGF), these treatments achieve a >90% probability of improving visual acuity by three lines on an eye chart after two years of treatment [10,11]. In contrast, dry AMD still lacks comparably effective treatment options, particularly in its advanced form, GA [12,13].

Recent breakthroughs have brought new hope for the treatment of GA. The U.S. Food and Drug Administration (FDA) has approved pegcetacoplan (Syfovre^®^) and avacincaptad pegol (Izervay), both of which target the complement system—crucial in GA progression. Pegcetacoplan inhibits complement component C3, whereas avacincaptad pegol blocks C5, significantly slowing GA lesion growth [14,15,16,17]. However, pegcetacoplan has not been approved by the European Medicines Agency (EMA), owing to concerns regarding safety and insufficient “clinically meaningful benefits” [18].

Restoring light sensitivity to degenerated retinas remains a central goal in AMD therapy. Traditional strategies such as gene therapy and cell transplantation face significant challenges regarding targeted delivery, long-term efficacy, and immune rejection of the graft. These obstacles have limited their application and slowed the translation of such therapies into clinical practice [19,20]. Gene therapy involves the delivery of a functional copy of a defective gene using a viral vector. This strategy led to the development of the first approved ocular gene therapy, voretigene neparvovec, which delivers the RPE65 photoisomerase gene to treat Leber congenital amaurosis. While this approach holds great promise for several monogenic inherited retinal disorders, it is not feasible for complex, polygenic or multifactorial conditions such as AMD [21,22]. Retinal prostheses, such as the Argus II system, have demonstrated the feasibility of restoring basic light perception and navigation in patients with profound vision loss; however, these devices are limited by low spatial resolution, complex surgical implantation, and variable long-term performance [23,24,25,26]. Pharmacological photoswitches represent another approach, offering the advantage of non-genetic, reversible modulation of retinal activity. Nonetheless, their effects are transient, requiring repeated administration [27,28,29]. Cell-based therapies, including transplantation of photoreceptor precursors or retinal pigment epithelial cells, hold promise for structural replacement, but integration and functional synaptic connectivity remain substantial [30,31,32].

Optogenetics presents a novel approach by introducing exogenous opsins into surviving retinal bipolar cells (BCs) and retinal ganglion cells (RGCs). These genetically modified cells can be activated by specific wavelengths of light amplified by external devices, effectively bypassing the lost photoreceptor and transmitting visual information to the brain [19,20]. Optogenetic approaches provide the potential for stable, long-term expression after a single administration; targeted activation of specific retinal cell populations; and compatibility with existing neural circuits. Over the last years, a growing body of research has supported that optogenetics could be a vision-restoring strategy in patients with retinal disease and well-preserved BCs and RGCs [20,33,34,35,36].

This review aims to provide a comprehensive overview of optogenetics in AMD research, covering its principles, preclinical applications, available optogenetic tools, gene delivery strategies, and the challenges of translating this technology into clinical therapies for AMD. This may highlight the current knowledge and future perspective for the feasibility of this alternative therapeutic approach in AMD.

## 2. Principles of Optogenetics

Optogenetics relies on the introduction of light-sensitive proteins, typically microbial opsins, into specific cell types within a living organism. In the context of retinal degeneration, the primary goal of optogenetic therapy is to render the remaining retinal neurons light-sensitive after photoreceptor loss. By expressing opsins in downstream neurons, such as RGCs or BCs, these neurons can be directly activated by light projected onto the retina, effectively replacing lost photoreceptor function and transmitting visual signals to the brain [34,36,37].

The strength of optogenetics lies in its ability to achieve cell-type specificity and temporal precision. Genetic targeting strategies using viral vectors with cell-specific promoters enable opsin expression in defined neuronal populations. Additionally, opsin-expressing cells can be activated with millisecond-scale precision using patterned light pulses [33,38].

Most of the current literature on optogenetic therapies has focused on inherited retinal disorders. Viral transduction, in particular, the use of adeno-associated viral (AAV) vectors, currently represents the most effective and clinically validated method for delivering optogenetic constructs to retinal cells [39,40]. In these studies, patients who were legally blind showed measurable improvements in visual function, including visual acuity and electrophysiological tests. However, the interpretation of these functional outcomes must be made with caution. Potential confounding factors, such as opacities associated with AAVs vectors, may affect visual outcomes. These include corneal keratic precipitates, vitreous haze, and cataract formation, all of which can independently impair vision and influence test results [41]. Despite the clinical predominance of AAV, other delivery systems have also been investigated. Lentiviral vectors allow for a larger packaging capacity but show limited penetration and distribution within the retina [42], while non-viral approaches such as electroporation and nanoparticle-based systems have demonstrated proof-of-concept efficacy in animal models [43,44]. Nevertheless, AAV vectors remain the most established and clinically validated strategy, as exemplified by the FDA approval of Luxturna^®^ for *RPE65*-associated retinal dystrophy [39,40].

### 2.1. AAV Serotypes and Delivery Mechanisms

Efficient and safe delivery of opsin genes to target retinal cells is critical for the success of optogenetic therapy. Viral vectors, particularly AAVs, are widely used, owing to their high transduction efficiency and low immunogenicity [22,45]. Table 1 summarizes the main natural and engineered AAV serotypes that have been employed for retinal gene delivery [39,40,46,47,48,49,50,51,52,53,54,55].

AAV-based vectors have been extensively investigated in both animal and human models, culminating in the first approved AAV gene therapy, voretigene neparvovec (Luxturna^®^), for Leber’s congenital amaurosis. Since then, this strategy has emerged as the preferred delivery method for optogenetic approaches and is currently being evaluated in clinical trials for retinitis pigmentosa [30]. Despite their advantages, several challenges complicate the use of AAV vectors in retinal gene therapy. Their limited packaging capacity (~4.7 kb) restricts application to smaller genes, thereby excluding many associated with inherited retinal diseases. In addition, variability in tropism among serotypes can limit efficient targeting of specific retinal cell types, while pre-existing neutralizing antibodies may reduce efficacy or prevent treatment in some patients. Long-term safety also remains a concern, as persistent transgene expression could provoke immune responses or off-target effects. A further limitation is the restricted penetration of AAV vectors into the inner retina after intravitreal delivery, largely due to barriers such as the internal limiting membrane, which reduces the transduction efficiency of deeper cell layers [56,57,58,59,60,61,62,63]. Advances in capsid engineering have enabled efficient transduction of the inner retinal layers. This has been achieved through several approaches, including the tyrosine-to-phenylalanine (Y–F) surface mutations that enhance intracellular trafficking; the development of multi-residue variants such as AAV2 (2–4YF); and the generation of the AAV2 (7m8) variant, which carries a seven-amino acid insertion in the VP1 capsid protein [49,54,60,64,65,66].

AAV serotypes exhibit varying tropism for different retinal cell types, allowing for targeted gene delivery through three main routes. Intravitreal, subretinal injections or suprachoroidal injections are the primary routes of administration for delivering AAV vectors to the retina [45]. Intravitreal injections are the safest and most widely used procedure; however, their effectiveness is limited by the vitreous and the internal limiting membrane (ILM), which act as a barrier. As a result, transduction is restricted to the inner retinal layers (e.g., retinal ganglion cells, inner nuclear layer), especially for serotypes like AAV-2, AAV-6, and AAV-8 [45,63]. ILM digestion techniques (like enzymatic treatment or sub-ILM injection) have shown substantial improvements in transduction efficiency to deeper retinal layers [22,63].

Subretinal injections have been demonstrated to reliably target photoreceptors and RPE with almost all the AAV phenotypes. However, it requires pars plana vitrectomy (PPV); creating a retinal detachment; and carries risks such as cataract formation, inflammation, and structural damage to the retina [45,63]. Suprachoroidal injections reduce the risk of ocular complications, avoiding PPV and subretinal surgery while enabling access to the outer retina without traversing the ILM or lens. The transduction efficiency may be limited by proteasomal degradation during transcytosis through the RPE and by clearance from the choriocapillaris and blood–retinal barrier. Variant capsids like AAV2tYF have demonstrated enhanced photoreceptor expression via this route [45,58,67,68]. In particular, engineered AAV2 capsids incorporating cell-penetrating peptides demonstrate improved penetration of the ILM. These peptides consist of short amino acid sequences, enriched in positively charged residues, which enhance interactions with negatively charged cell membranes and thereby facilitate more efficient penetration [69]. Electric micro-currents, so-called electric-current vector mobility (ECVM), also improve the transduction of AAV-vector following intravitreal administration, potentially causing a possible upregulation of basic fibroblast growth factor from Müller cells [70]. Another mechanism for increasing retinal transduction involved the binding of AAV to heparan sulfate proteoglycans [71]. See Table 2 for further details.

Both subretinal and intravitreal injection routes have demonstrated robust transgene expression, as evidenced by strong fluorescence signals in experimental mouse models [72]. Subretinal delivery produced a more intense and localized fluorescence; the intravitreal delivery resulted in a broader distribution of transduction across the retina [73].

While AAV vectors remain the most extensively studied platform for ocular gene delivery, alternative methods have also been investigated. These include lentiviral vectors, which can accommodate larger genetic payloads but show limited retinal penetration [74,75]. Non-viral approaches, such as nanoparticles, have also been evaluated for delivering drugs and genes to the retina. These systems provide several advantages, including low immunogenicity, larger cargo capacity, and the potential for sustained expression, making them attractive candidates for retinal gene therapy applications [76,77,78,79]. Other non-viral vectors include naked DNA, siRNA, mRNA, miRNA, liposomes, niosomes, as well as polymers and inorganic complexes. These approaches offer advantages in terms of safety and reduced immunogenicity but are generally limited by lower stability, reduced transfection efficiency, and less specificity compared to viral vectors [80].

### 2.2. Optogenetic Light Sources

External light sources are required to activate expressed opsins. These systems must be designed to deliver safe and effective light intensity and wavelengths to the retina. Recent advances in light-emitting-diode (LED) microdisplay technology and optogenetic prostheses now enable precise, patterned light projection onto the retina, stimulating opsin-expressing cells and generating artificial vision [81].

### 2.3. Opsin Variants for AMD Therapy

Opsins are transmembrane proteins that function as light-gated ion channels or pumps. These light-sensitive proteins can mediate either depolarization or hyperpolarization. Light-activated cation channels induce depolarization and neuronal firing. In contrast, chloride pumps cause hyperpolarization and neuronal inhibition.

Their properties influence visual restoration outcomes [30,37,44,56,64,66,79,82,83,84,85,86,87,88]:

- Sensitivity: Highly sensitive opsins allow activation with low light intensities, minimizing phototoxicity and maximizing compatibility with ambient light conditions. Next-generation opsins engineered for low light intensity thresholds are continuously being developed.
- Kinetics: The speed of opsin activation and inactivation influences the temporal resolution of the restored vision. Faster kinetics may be crucial for processing rapidly changing visual information.
- Spectral Properties: Different opsins are activated by different wavelengths of light. Choosing opsins with activation spectra that are well transmitted through ocular tissues and can be safely delivered by external light sources is important. Red-shifted opsins reduce phototoxicity, the thermal effect, and oxidative stress associated with high-intensity illumination. These properties help minimize the risk of retinal damage related to light stimulation [38,88].
- Expression Stability: Robust and stable expression ensures long-term therapeutic efficacy.

Optogenetic research has progressed from Channelrhodopsin-2 (ChR2) to enhanced variants like ChrimsonR, ChRmine, and MCO1, which improve light sensitivity and reduce phototoxicity [37]. Table 3 summarizes the main opsins investigated in optogenetic strategies and their primary cellular targets [37].

Currently, intraocular injection remains the standard method for delivering AAV-based gene therapies, as it enables targeted delivery and efficient transduction of retinal cells [46,63,82,89,90,91,92]. While the need for ocular injections may raise concerns about patient compliance, it is worth noting that intravitreal injections are already a routine and widely accepted clinical practice for the administration of anti-VEGF therapies in neovascular AMD [67,89,90,91,92,93,94]. Nevertheless, efforts are underway to develop less invasive delivery methods, including nanoparticle- and polymer-based non-viral vectors, suprachoroidal and subretinal delivery devices [70,72,76,78,95], and engineered AAV variants capable of crossing the blood–retinal barrier [52,69]. Although these strategies remain experimental, they offer promising avenues to enhance patient comfort and broaden the applicability of gene therapy in the future.

### 2.4. Cellular Targets

One of the most important aspects of optogenetic therapy concerns the choice of cellular target, which is likely to influence the quality of restored vision. Two major cellular targets are represented by retinal BCs and RGCs [73,83,84,96,97,98,99]. Targeting BCs is considered advantageous because they represent the first relay in the inner retina, preserving more of the native retinal signal processing. Several studies have demonstrated the successful transduction of ON-bipolar cells using AAV vectors engineered with optimized capsids and cell-specific promoters, such as the Grm6 enhancer [73,96,97]. These strategies have enabled high-level expression of optogenetic actuators like channelrhodopsins in BCs, inducing light-evoked responses and restoring visual behaviors in blind animal models. Moreover, the bipolar cell layer allows a finer level of spatial resolution compared with downstream targets and may support higher-order visual processing.

RGCs, although located further downstream in the visual pathway, are also effective targets for optogenetic therapy, especially when the inner retinal layers are intact, but bipolar cell transduction is limited. Expression of light-sensitive proteins such as channelrhodopsin, melanopsin, or cone opsins in RGCs has successfully restored visual responsiveness and behavioral light perception in rodent models of retinal degeneration. Notably, Cehajic-Kapetanovic et al. [96] showed that ectopic expression of human rod opsin in both RGCs and BCs restored physiological and behavioral light responses at relatively low light intensities, supporting potential translation into clinical therapies. Similarly, Berry et al. [83] demonstrated that medium-wavelength cone opsin (MW-opsin) expressed in RGCs restored both light sensitivity and adapting vision, enabling object recognition under natural lighting conditions. This was hypothesized to represent a significant advancement over traditional microbial opsins, which lack adaptation and require high-intensity stimulation.

Ultimately, the selection between targeting bipolar cells or RGCs may depend on several factors, including the stage of disease progression, degree of retinal remodeling, vector accessibility, and desired visual outcomes. BCs may offer better integration with residual retinal circuits and more naturalistic vision, whereas RGCs may represent a more feasible target in severely degenerated retinas where upstream retinal architecture is compromised. Ongoing research continues to explore hybrid strategies and refined targeting to maximize both safety and efficacy in future clinical applications [98,100].

### 2.5. Clinical Evidence

Optogenetic research has progressed from Channelrhodopsin-2 (ChR2) to enhanced variants like ChrimsonR, ChRmine, and MCO1, which improve light sensitivity and reduce phototoxicity [37].

In 2021, the first clinical report described a retinitis pigmentosa patient treated with optogenetics via AAV2-ChrimsonR gene therapy. After training with light-stimulating goggles, the patient regained object perception [101]. The clinical trials RESTORE and STARLIGHT have investigated MCO1 therapy under ambient light, showing improvements in visual acuity without severe adverse effects [88,102,103,104,105].

Studies on vertebrate opsins like human Rhodopsin (Rho) and Melanopsin (OPN4) have demonstrated high intrinsic light sensitivity and biocompatibility, reducing immunogenicity risks [96,106,107]. Chimeric proteins such as GHCR (Gleobacter–Human Chimeric Rhodopsin) and SNAG-mGluR2 offer enhanced optogenetic function in mouse models, supporting realistic perceptions under natural lighting conditions [84,96,97,98,99,100,101,104,105,106,107,108,109,110].

## 3. Preclinical Applications of Optogenetics in AMD Models

While no animal model fully replicates all aspects of human AMD, several models exhibiting features of photoreceptor or RPE dysfunction have been used to evaluate optogenetic therapies. Genetic models of retinal degeneration were developed with mutations in genes linked to photoreceptor degeneration, exhibiting a progressive decline in light sensitivity. Rodent models with rd1 and rd10 mutations treated with MCO-010 optogenetic therapy have demonstrated stabilization in retinal thickness, indicating a reduced cellular loss of the remaining photoreceptors, as well as increased light sensitivity [111,112]. Studies in these models show that expressing opsins in RGCs or bipolar cells can evoke light responses and induce behavioral improvements, indicating functional vision restoration [56,82,84,113,114,115,116,117,118,119].

In addition to genetic models, chemically induced models of retinal damage have been used. For instance, intravitreal injection of agents like sodium iodate selectively damages the RPE, mimicking pathogenic features observed in GA. These models contribute valuable insights for optogenetic research in the setting of dry AMD [120,121,122].

Moreover, laser-induced choroidal neovascularization (CNV) models offer insights in the neovascular form of AMD that may affect the outer retina and RPE due to exudative and fibrotic changes. These models are useful to understand the effect of optogenetic strategies in restoring vision [123].

Preclinical studies across these models consistently demonstrate that optogenetic stimulation can induce light responses in targeted retinal neurons, leading to measurable improvements in electrophysiological and behavioral tests.

### Clinical Considerations for Optogenetic Therapy in AMD

Most research on AMD and optogenetics focuses on GA, the advanced dry AMD stage. Studies indicate that inner retinal layers are well preserved in 83.7% of GA patients (SD-OCT analysis) and 74.3% of histopathology samples, supporting optogenetics viability in this group [56,57].

The suitability of optogenetic therapy varies across AMD stages, as well as the integrity of inner retinal layers. In early-stage AMD, the retinal architecture is more preserved, particularly the inner retinal layers, such as bipolar and ganglion cells. This preservation creates a favorable environment for optogenetic intervention, as these inner retinal cells represent the main targets to bypass damaged photoreceptors while still enabling the transmission of visual information to the brain, using the remaining neuronal cells to achieve photosensitivity [82]. Insights from retinal degeneration models have shown that inner retinal remodeling emerges only after the outer nuclear layer has been largely depleted of rods and cones, with Müller cell processes forming a seal between the remnant retina and the choroid or surviving retinal pigment epithelium. Once initiated, remodeling follows a stereotyped sequence across different models and includes neuronal loss, relocation of amacrine and bipolar cells, fragmentation of the inner plexiform layer, neurite sprouting, and the formation of ectopic synaptic foci or “microneuromas.” Additional features include hypertrophy and migration of Müller cells, vascular invasion from both vitreal and choroidal sides, and even RPE cell ingrowth into the neural retina. These profound structural and synaptic alterations highlight the complexity of rewiring in advanced degeneration and underscore the challenge of integrating optogenetically reactivated neurons into reorganized retinal circuits [124,125,126].

The presence of subretinal drusenoid deposits (SDDs) or reticular pseudodrusen (RPD) can represent a relevant factor to consider, as it has been demonstrated greater retinal thickness changes in these eyes. These changes are even more remarkable in the inner retina for newly formed RPD, a structural alteration that is also reflected in retinal sensitivity measured through microperimetry [127,128,129]. Notably, the ganglion cell layer was found to be thinner in both patients affected by RPD alone or by RPD with outer retina atrophy [128]. Therefore, SDD/RPD may represent a key factor affecting the optogenetic procedure and likely the functional outcomes.

**Figure 1:**
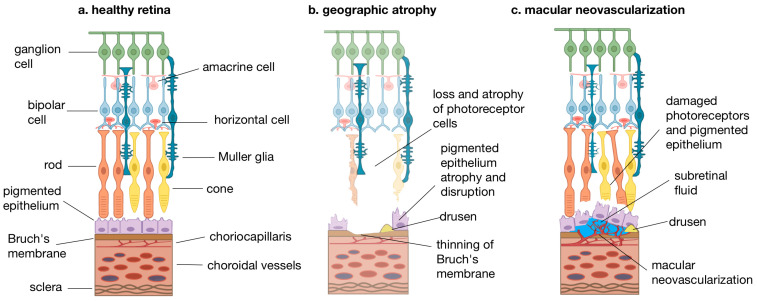
Structural comparison of retinal layers in healthy retinas versus advanced forms of age-related macular degeneration (AMD). (**a**) Healthy retina: Normal retinal architecture showing organized layers of ganglion cells, bipolar cells, rods, cones, and Müller glia overlying the retinal pigment epithelium (RPE), Bruch’s membrane, and choroidal vasculature. (**b**) Geographic atrophy (GA): Characterized by the loss and atrophy of photoreceptor cells, disruption and atrophy of the RPE, presence of drusen, and thinning of Bruch’s membrane. These changes are hallmarks of advanced dry AMD. (**c**) Macular neovascularization (MNV): Representative of the neovascular (wet) form of AMD, showing damaged photoreceptors and RPE, subretinal fluid accumulation, and invasion of abnormal blood vessels from the choroid into the subretinal space (macular neovascularization).

In contrast, late-stage AMD can present a more relevant retinal anatomical disruption. In GA, a thinning of the outer nuclear layer (ONL) co-localizes with atrophic zones, reflecting a photoreceptor loss [127,130,131]. However, a minimal decrease in the ganglion cell layer volume of about 16% has been described in patients with GA [132]. In the setting of MNV, several factors may adversely impact the integrity of the inner retina. These include the presence of subretinal hemorrhage, fibrotic tissue formation, intraocular pressure modifications due to anti-VEGF procedures, and the cumulative effects of repeated intravitreal anti-VEGF therapies, all of which may contribute to structural or functional alterations of inner retinal layers [133]. Moreover, the ganglion cell thickness has been found to be a prognostic factor strongly associated with visual recovery in eyes with MNV secondary to AMD [134]. See Figure 1 for further details.

Given these differences, patient selection criteria for optogenetic trials must account for retinal integrity and inner retinal layer preservation. Patient selection should also be performed with caution, taking into account additional factors that may compromise the integrity of the inner retina. A relevant example is the co-occurrence of glaucoma, which can lead to progressive degeneration of retinal ganglion cells and may significantly limit the efficacy of optogenetic therapies targeting these cells [135].

## 4. Challenges and Future Directions

Despite encouraging outcomes from preclinical studies, several critical challenges remain before optogenetics can become a widely applicable therapeutic strategy for AMD. One of the foremost obstacles is achieving cell-type specificity, which involves the precise and stable expression of opsins in the intended retinal cells. Off-target expression poses a significant risk, as it may lead to unintended cellular responses and potential adverse effects [136,137].

Another major limitation is the resolution of the restored vision. At present, optogenetic techniques tend to produce low-resolution artificial visual experiences. Therefore, significant efforts are needed to refine opsin properties and improve the design of light stimulation methods to enhance visual acuity and image clarity [37,87,138].

Long-term safety and stability also represent critical considerations. The sustained expression of opsins and the long-term effects of gene delivery systems must be carefully evaluated, particularly concerning potential immune responses and retinal toxicity over time [79].

Finally, the integration of optogenetically activated neurons with existing retinal circuits is essential for generating meaningful and functional visual perception. A critical challenge remains in the context of advanced retinal degeneration, where cell loss and structural remodeling may hinder efficient transduction and functional recovery. Consequently, therapeutic outcomes are likely to be more favorable in less degenerated or earlier disease stages [30,64]. A deeper understanding of how these modified neurons communicate with residual retinal pathways will be fundamental to translating optogenetic vision into a coherent and useful experience for patients.

## 5. Future Research Focus

To enhance the effectiveness of optogenetic therapy for AMD, future research is focusing on several key areas. One major direction involves the development of advanced opsins—light-sensitive proteins that can be engineered to possess greater light sensitivity, faster activation and deactivation kinetics, and optimized spectral properties tailored to the ambient light conditions of the human retina. Enhancing these characteristics can significantly improve visual restoration outcomes.

Another essential area of progress lies in improving gene delivery methods. Researchers are working on developing novel AAV serotypes that offer targeted delivery to specific retinal cells while maintaining a favorable safety profile. These vectors aim to overcome current barriers to efficient transduction and reduce immune responses, making long-term treatment more viable.

In parallel, efforts are being directed toward designing high-resolution stimulation systems. These include smart visual prostheses and optical devices that can precisely deliver light stimuli to genetically modified retinal neurons. Such systems are critical for translating the cellular-level responses induced by optogenetic activation into meaningful visual perception for the patient. There is also growing interest in combination therapies that integrate optogenetics with other treatment strategies. These may include gene replacement therapies or the use of neuroprotective agents aimed at preserving retinal structure and function. By combining modalities, researchers hope to achieve synergistic effects that enhance overall therapeutic outcomes.

At present, no clinical trials have been registered specifically for optogenetic therapy in AMD. Most ongoing clinical studies are focused on inherited retinal diseases, such as retinitis pigmentosa. Nonetheless, the rapid pace of advancements in gene therapy, device development, and retinal neuroprotection is paving the way for future clinical applications in AMD. This growing body of research holds promise for translating optogenetic strategies into viable treatments for patients with this widespread and currently incurable condition.

## 6. Conclusions

Optogenetics represents a novel therapeutic strategy for vision restoration in age-related macular degeneration. Preclinical studies demonstrate the feasibility of using light-sensitive opsins to activate surviving retinal neurons and elicit functional visual responses.

Ongoing research in optogenetic tools, gene delivery techniques, and light stimulation systems is paving the way for clinical applications. Moreover, while optogenetic therapy holds promise, its efficacy may depend strongly on the stage of disease and the extent of remodeling present. Although significant challenges remain, technological progress offers hope for AMD patients suffering from severe vision loss. Future clinical trials will be crucial in assessing the safety, efficacy, and practicality of optogenetic therapy in AMD management.

## Figures and Tables

**Table 1:** Natural and engineered AAV serotypes used for retinal gene delivery.

AAV Serotype/Variant	Key Features	Delivery Route	Reference
AAV2	Most widely used in retinal gene therapy; limited penetration across ILM	Subretinal/Intravitreal	Maguire et al., 2008 [39]
AAV2-RPE65 (Luxturna)	First FDA-approved ocular gene therapy; durable expression	Subretinal	Jacobson et al., 2015 [40]
AAV5	Altered tropism through capsid modifications	Subretinal	Auricchio et al., 2001 [46]
AAV8	Efficient transduction; tested in ocular models	Subretinal	Natkunarajah et al., 2008 [47]
AAV1–9	Systemic injection shows broad tissue tropism	Systemic	Zincarelli et al., 2008 [48]
AAV2.7m8	Directed evolution variant optimized for intravitreal delivery	Intravitreal	Dalkara et al., 2013 [49]
AAV variant (Müller selective)	Efficient intravitreal transduction of Müller cells	Intravitreal	Klimczak et al., 2009 [50]
Anc80	Potent synthetic capsid with broad tropism	Systemic/Intravitreal	Zinn et al., 2015 [51]
Engineered capsid	Noninvasive delivery to foveal cones	Intravitreal	Khabou et al., 2018 [52]
Gene-editing AAV approach	CRISPR-based therapy for LCA10	Subretinal	Maeder et al., 2019 [53]
Tyrosine-mutant AAV2	Improved transduction efficiency in mouse retina	Subretinal	Petrs-Silva et al., 2011 [54]
Cre-dependent AAV variants	Designed for widespread CNS delivery, tested in eye models	Intravitreal/Systemic	Deverman et al., 2016 [55]

Natural serotypes such as AAV2, AAV5, AAV8, and AAV9 have been used extensively in preclinical and clinical studies, with AAV2 serving as the basis for the first FDA-approved ocular gene therapy. Engineered variants, including AAV2–7m8, ShH10, Anc80L65, AAV2-NN/GL, and AAV8BP2, have been designed to enhance transduction efficiency, bypass the inner limiting membrane barrier, or target specific retinal cell types. Some systemic AAV9 derivatives, such as PHP.B and PHP.eB, demonstrate enhanced CNS tropism in rodent models but show limited efficacy in primates. The delivery route column emphasizes how each vector is preferentially administered to achieve optimal transduction. Abbreviations: AAV—Adeno-associated virus; RPE—Retinal pigment epithelium; ILM—Inner limiting membrane; CNS—Central nervous system; FDA—U.S. Food and Drug Administration.

**Table 2:** Comparison of AAV delivery routes for retinal gene therapy.

Injection Route	Target Cells	Advantages	Limitations and Risks
Intravitreal (IVT)	Inner retina (RGCs, Müller cells)	Safest and office based	Low outer retina transduction; ILM/vitreous barriers; potential neutralization/inflammation
Subretinal (SR)	Photoreceptors, RPE	Highest efficiency targeting RPE and PRs	Requires PPV and detachment; surgical complexity; risk of retinal damage
Suprachoroidal (SC)	Outer retina (ONL, RPE)	Less invasive; avoids ILM and PPV	Proteasomal degradation; blood–retinal barrier; variable efficiency; potential mild immune response

Abbreviations: AAV—Adeno-Associated Virus; IVT—Intravitreal Injection; SR—Subretinal Injection; SC—Suprachoroidal Injection; RGCs—Retinal Ganglion Cells; PRs—Photoreceptors; RPE—Retinal Pigment Epithelium; ONL—Outer Nuclear Layer; ILM—Inner Limiting Membrane; PPV—Pars Plana Vitrectomy.

**Table 3:** Main opsins investigated in optogenetic therapy and their primary cellular targets.

Cellular Target	Opsins	Notes
	**Microbial (channel opsins)**	
RGCs, ON BC	Channelrhodopsin-2 (ChR2)	First-generation microbial opsin
RGCs	ChrimsonR, ChRmine	Red-shifted variants with higher light sensitivity, lower phototoxicity
BC	MCO1	Multicharacteristic opsin, tested in RESTORE and STARLIGHT trials
	**Mammalian (GCPR opsins)**	
RGCs ON BC	Human rod opsin (RHO)	High intrinsic light sensitivity
RGCs	Melanopsin (OPN4)	Endogenous opsin; reduced immunogenicity risk
RGCs	Medium-wavelength cone opsin (MW-opsin)	Restores adapting vision under natural light conditions, high light sensitivity, and fast response kinetics
	**Chimeric/Engineered**	
RGCs	GHCR	Chimeric opsin (Gloeobacter–Human Chimeric Rhodopsin) with enhanced sensitivity and improved kinetics
RGCs	SNAG-mGluR2	Engineered opsin based on metabotropic glutamate receptor; modulates inhibitory signaling pathways

Abbreviations: mGluR2—metabotropic glutamate receptor 2; RGCs—Retinal Ganglion Cells; BC: bipolar cell.

## Data Availability

Not applicable.

## Abbreviations

The following abbreviations are used in this manuscript:

AMD	Age-related Macular Degeneration
AAV	Adeno-Associated Virus
AAV2	Adeno-Associated Virus Serotype 2
BCs	Bipolar Cells
ChR2	Channelrhodopsin-2
ChRmine	Channelrhodopsin Mine
CNV	Choroidal Neovascularization
FDA	U.S. Food and Drug Administration
GA	Geographic Atrophy
GHCR	Gleobacter–Human Chimeric Rhodopsin
ILM	Inner Limiting Membrane
IVT	Intravitreal Injection
LED	Light-Emitting Diode
MCO1/MCO-010	Multicharacteristic Opsin 1/010
MNV	Macular Neovascularization
MW-opsin	Medium-Wavelength Cone Opsin
ONL	Outer Nuclear Layer
OPN4	Melanopsin
PPV	Pars Plana Vitrectomy
PRs	Photoreceptors
RGCs	Retinal Ganglion Cells
Rho	Rhodopsin
RPE	Retinal Pigment Epithelium
RPD	Reticular Pseudodrusen
SC	Suprachoroidal Injection
SD-OCT	Spectral Domain Optical Coherence Tomography
SDD	Subretinal Drusenoid Deposits
SNAG-mGluR2	Synthetic Opsin–Metabotropic Glutamate Receptor 2 Fusion Protein
SR	Subretinal Injection
VEGF	Vascular Endothelial Growth Factor
